# Sacral neuromodulation to treat voiding dysfunction in patients with previous pelvic surgery for deep infiltrating endometriosis: our centre's experience

**DOI:** 10.1007/s00192-020-04478-z

**Published:** 2020-08-15

**Authors:** Marco Agnello, Mario Vottero, Paola Bertapelle

**Affiliations:** 1 Università degli Studi di Torino, Torino, Italy; 2 SC Urologia U, A.O.U. Città della Salute e della Scienza, Molinette Hospital (Corso Bramante 88, Torino – CAP 10126), Torino, Italy; 3 SC Neuro-Urologia, A.O.U. Città della Salute e della Scienza, Torino, Italy

**Keywords:** Sacral neuromodulation, Endometriosis, Urinary retention, Pelvic pain

## Abstract

**Introduction and hypothesis:**

Voiding symptoms/dysfunctions (VS/Ds) after surgery for deep-infiltrating endometriosis (DIE) are frequent (20% of patients) and, together with bowel dysfunctions, may represent a de novo disorder due to surgical damage of the pelvic plexus or a worsening of pre-existent functional damage. Sacral neuromodulation (SNM) might improve voiding symptoms by treating dysfunctional voiding. The aim of this study is to report our experience with SNM in patients treated with surgery for DIE.

**Methods:**

We retrospectively enrolled 13 patients with VS/Ds after surgery for DIE. All patients were investigated with urodynamic studies (UDS) and agreed to undergo SNM. Pre-existing VS/Ds, bowel disorders and pelvic pain, DIE surgical procedures, UDS and SNM test results were recorded.

**Results:**

After surgery for DIE, functional bladder outflow obstruction and detrusor acontractility were observed in nine and four patients, respectively. Chronic pelvic pain was present in seven cases. Twelve patients developed constipation, whilst one patient had de novo faecal incontinence. After the SNM testing period, nine patients (69.2%) experienced a significant improvement of symptoms that led to definitive implant. Four patients (30.8%) had no symptom relief and the system was removed.

**Conclusions:**

Functional bladder outflow obstruction and urinary retention are the most common VS/Ds after surgery for DIE. SNM may be an effective option for these patients, probably due to its action in improving the dysfunctional voiding, which was likely to be already present as part of the “endometriotic syndrome” and got worse after pelvic surgery. Results for pelvic pain control and gastrointestinal disorders should not be underestimated.

## Introduction

Endometriosis is defined as the presence of functional endometrial glands and stroma outside the uterine cavity, predominantly, but not exclusively, in the pelvic compartment. It is an oestrogen-dependent chronic inflammatory condition that affects 5–10% of women in their reproductive life and is associated with pelvic pain and infertility [1]. The most common locations for ectopic endometrial implants are the ovaries, parametrium, recto-vaginal septum, uterosacral ligaments and posterior cul-de-sac.

The term “deep-infiltrating endometriosis” (DIE) should be reserved for lesions in the retroperitoneal tissue (i.e., at sites different from those of “ordinary” lesions) but, for practical purposes, it includes lesions penetrating anatomical pelvic structures, mainly of the posterior compartment (93.4% of cases) [2], such as the utero-sacral ligaments, bowel, bladder, vaginal wall and ureters. These lesions may greatly alter the quality of life because of severe pain at menstruation and intercourse and pose particularly difficult surgical problems [3]. DIE is thought to affect 20% of patients with endometriosis. Due to the poor efficacy of medical treatments, symptomatic women affected by DIE are advised to undergo surgery.

Urinary symptoms (such as dysuria and painful voiding, hesitancy, intermittent flow, increase in daytime frequency, urgency and urge incontinence, alterations of bladder sensitivity, gross haematuria) are present—depending on the methods and questionnaires used to highlight these conditions—in 5–20% of patients suffering from endometriosis, before any kind of surgery, but are often masked by the prevalence of gynaecological and gastrointestinal disorders, particularly in the deep-infiltrating forms [4].

Following surgery for DIE, > 20% of patients experience voiding symptoms (dysuria, hesitancy, incomplete bladder emptying sensation), mainly related to urinary retention and dysfunctional voiding. These voiding symptoms/dysfunctions (VS/Ds) may represent a de novo disorder related to surgical damage of the pelvic plexus or worsening of pre-existent functional damage [4–8].

A few therapeutic alternatives for patients with persistent VS/Ds after surgery for DIE are available. Intermittent self-catheterisation remains the gold standard to treat urinary retention, to avoid recurrent urinary tract infections and urogenital prolapse due to abdominal straining. Dysfunctional voiding, which is related to poor relaxation of the external urethral sphincter (EUS), is thought to cause reflex inhibition of detrusor contraction by potentiating a spinal pro-continence reflex. The use of muscle-relaxant drugs to inhibit urethral sphincter activity has not been validated [9], and few results have been derived from intrasphincter botulinum toxin injection [10].

Sacral neuromodulation (SNM), consisting of continuous electrical stimulation of the sacral segmental unilateral nerve S3, enables bladder emptying by peripheral (inhibition of guarding reflex) and central action [11]. SNM has been recently suggested as an alternative to self-catheterisation in VS/Ds [12] and has been validated for the management of chronic idiopathic urinary retention refractory to medical therapies. Moreover, SNM might block urethral afferent activity allowing restoration of bladder sensation, detrusor contraction and voiding in patients with dysfunctional voiding [13], even if poor relaxation of the EUS seems to persist despite successful voiding post treatment [14], suggesting that patients void due to the return of a forceful contraction rather than a direct efferent effect on sphincter relaxation.

However, few case series and validated data on SNM in the treatment of VS/Ds after surgery for DIE are available.

The aim of this preliminary study is to report our experience with SNM in patients suffering from VS/Ds who came to urologist’s attention for the first time after surgery for DIE.

## Materials and methods

We retrospectively enrolled 13 patients who underwent a SNM implant from April 2015 to March 2019 for persistent VS/Ds after pelvic surgery for deep-infiltrating endometriosis. All patients underwent invasive urodynamic studies (UDS) supplemented with needle electromyography of the external urethral sphincter (EUS EMG) before SNM.

The study was conducted according to the Declaration of Helsinki general principles for medical research involving humans. Following our Institutional Ethical Committee Approval (protocol no. 0036753) and acquisition of informed consent, patient data were obtained (both from outpatient visit and previous medical records) and collected in the Sacral Neuromodulation Database of our institution. We used SPSS software to analyse data and perform our computations.

According to the International Continence Society (ICS) recommendations [15], a bladder outflow obstruction due to overactive urethral function during voiding (dysfunctional voiding) is considered when all the following parameters are observed: a maximum flow rate < 15 ml/s; a maintained detrusor contraction (> 25 cmH_2_O) in the presence of incomplete bladder emptying (post-voided volume > 100 ml and voided volume > 200 ml); an increase in EUS EMG activity during micturition [16]. Bladder acontractility was diagnosed when a complete absence of detrusor pressure waves was observed on pressure-flow studies (PFS), regardless of the post-voided residual and maximum flow rate. Cystometry was performed in the sitting position through a double-lumen catheter (6 Ch). The filling rate was 30 ml/min. Filling was stopped when the cystometric volume of 300 ml was reached to avoid bladder overdistension. At the end of the filling phase, the patient was asked to void, and the detrusor contraction was recorded if micturition was obtained. EUS EMG was performed with the Albyn Medical AROS System©, after positioning of the needle with the aid of the Keypoint Medtronic® System, according to ICS Supplementary Urodynamic Tests (ICS-SUT) with EMG recommendations [17].

Only three patients were investigated before the SNM test for pudendal neuropathy using bilateral pudendal nerve terminal motor latency (PNTML) and external anal sphincter electromyography (EAS EMG) studies. In PNTML evaluation, the delay between the electrical stimulation of the pudendal nerve and the EMG activity of the external anal sphincter (EAS) was recorded using a St. Mark’s glove electrode©. During EAS EMG, EMG activity was recorded by an EMG needle inserted in the four quadrants of the EAS.

The SNM Interstim© staged procedure was performed using a standardised electrode placement technique [18]. In the first stage, a permanent tine lead was implanted under local anaesthesia with fluoroscopic control into the S3 sacral foramen, on the left or right side, considering the best motor and sensory response at the lowest amplitude threshold in the testing phase. In the second stage, a permanent pulse generator was implanted for the patients with significant objective relief of symptoms after 6–8 weeks of the testing period. In those with negative symptom relief, the lead and external temporary extensions were removed under local anaesthesia.

VS/Ds before SNM therapy, previous DIE surgical procedures, UDS results, time between surgery and SNM test, age at the SNM test, SNM test results and daily number of self-catheterisations (if performed) before the SNM test and after permanent implantation of the system were recorded for each patient.

## Results

Patient characteristics are reported in Table 1.

**Table 1:** Patient characteristics. Surgical, clinical and SNM information are reported

Surgical information					Clinical status before SNM, assested by urodynamic and neurophysiological tests						Sacral neuromodulation results		
ID	Endometriosis localisations	Number of surgeries for endometriosis	Annessiectomy	Intestinal resection	Bladder function	Self-catheterisation (N daily)	Intestinal function	Trans-anal irrigation	Pelvic pain	PNTML and EAS EMG	Year of implant	SNM benefits	SNM removal
1	Utero-sacral ligaments; rectum; vagina	1	Not performed	Performed	Bladder acontractility	Yes (5)	Constipation	Yes	No		2017	Improvement in bladder emptying, reduction from 4 to 2 daily catheters	No
2	Uterus; ovary	1	Performed, bilateral	Not performed	Functional obstruction	Yes (4)	Faecal incontinence	No	Yes		2019	Total resolution of bladder and pelvic pain	No
3	Utero-sacral ligaments; rectum	1	Not performed	Not performed	Functional obstruction	No	Constipation	No	Yes		2017	Increase in bladder sensitivity and pain control	No
4	Peritoneum; left ureter	4	Not performed	Not performed	Functional obstruction	No	Constipation	No	Yes	Normal	2017	**No benefits**	**Yes**
5	Utero-sacral ligaments; rectum	4	Not performed	Performed	Functional obstruction	No	Constipation	No	No		2015	**No benefits**	**Yes**
6	Uterus; vagina; sigma	1	Not performed	Performed	Functional obstruction	Yes (4)	Constipation	No	No	Bilateral pudendal neuropathy	2015	**No benefits**	**Yes**
7	Utero-sacral ligaments	3	Not performed	Not performed	Functional obstruction	No	Constipation	No	Yes	Bilateral pudendal neuropathy	2018	Increase in bladder sensitivity and pain control	No
8	Utero-sacral ligaments; rectum	1	Not performed	Not performed	Bladder acontractility	Yes (4)	Constipation	No	Yes		2018	Bowel constipation improvement	No
9	Utero-sacral ligaments; rectum	1	Not performed	Performed	Functional obstruction	Yes (4)	Constipation	No	No		2015	Increase in bladder sensitivity and pain control	No
##	Utero-sacral ligaments; ovary; rectum	2	Not performed	Performed	Functional obstruction	No	Constipation	No	Yes		2016	Increase in bladder sensitivity and pain control	No
##	Parametrium, vagina, rectum, sacral plexus, right pudendal nerve	1	Not performed	Performed	Bladder acontractility	Yes (5)	Constipation	No	No		2015	**No benefits**	**Yes**
##	Douglas pouch	1	Not performed	Not performed	Functional obstruction	No	Constipation	No	No		2017	Increase in bladder sensitivity and pain control	No
##	Utero-sacral ligaments, recto-vaginal septum	1	Not performed	Not performed	Bladder acontractility	Yes (4)	Constipation	No	Yes		2015	Improvement in bladder emptying, reduction from 4 to 1 daily catheter	No

Diagnoses of bladder acontractility and functional obstruction were made used standardised criteria described in the Materials and Methods section. Constipation is considered if < 3 bowel movements per week are reported. PNTML = pudendal nerve terminal motor latency. EAS EMG = external anal sphincter electromyography. SNM = sacral neuromodulation

Surgery for deep-infiltrating endometriosis involved the uterus, ovaries, utero-sacral ligaments, parametrium, recto-vaginal septum, Douglas pouch and peritoneum in most cases. Bilateral adnexectomy was performed in one case, and partial intestinal resection was performed in six cases.

The main voiding dysfunction after surgery, assessed by UDS and EUS EMG and observed in nine patients (69.2%), was dysfunctional voiding due to overactive urethral function, defined as described above. Three out of nine patients with an outflow obstruction needed self-catheterisation (4 catheters per day) to complete bladder emptying. Main symptoms in this cohort of patients were dysuria, hesitancy, intermittent flow and incomplete bladder-emptying sensation. Bladder acontractility was observed in the remaining four patients (30.8%), for whom self-catheterisation was mandatory (4–5 catheters per day).

Half of the patients with bladder acontractility underwent an intestinal resection during surgery for DIE.

Intestinal constipation was present in 12 cases (92.3%), with use of trans-anal irrigation in only one patient. Faecal incontinence after surgery was observed in one case (7.7%).

Pelvic pain was present in seven patients (53.8%) after surgery (mean VAS score: 7.5).

Bilateral pudendal neuropathy was recorded in two out of three patients who underwent neurophysiological studies.

After the SNM testing period, nine patients (69.2%) experienced a significant improvement of symptoms that led to definitive implant. As shown in Table 1, benefits from SNM were different. Two patients, with initial diagnosis of bladder acontractility, voided spontaneously and reduced the number of daily catheterisations from four to one and two, respectively. Only self-intermittent catheterisations with a post-voided residual > 100 ml were maintained. Five patients experienced better pain control (mean VAS score 7.5 to 4 after SNM implant) and onset of bladder sensitivity that helped timing of catheterisation to avoid bladder overdistension (residual > 500 ml per catheterisation). One patient developed an improvement of intestinal constipation, with regularisation of defecatory habits, and one patient declared an almost total resolution of anal and pelvic pain (VAS score 8 to 2 after SNM implant).

Four patients (30.8%) had no symptom relief and the quadripolar tined-electrode was removed, including one of the two patients with the previously identified pudendal neuropathy.

## Discussion

Pelvic surgery for deep-infiltrating endometriosis is frequently associated with VS/Ds, mainly because of incomplete bladder emptying and increased post-voided residual.

On one hand, these symptoms could be justified by the aggressive surgical approach often adopted and the partial or total lesion of the inferior hypogastric and pelvic plexus, which lead to a hypo-acontractile bladder, with subsequent episodes of partial or total urinary retention. Ballester et al. showed that the rate of significant post-voiding residual volume after colorectal resection for endometriosis ranges from 15 to 20%. This rate seems to be higher (up to 30%) after proximal utero-sacral ligament resection. This might be explained by the location of the inferior hypogastric plexus at the proximal portion of the utero-sacral ligaments [19]. Moreover, postoperative VS/Ds requiring self-catheterisation are significantly related to partial colpectomy, as proven by Zilberman et al. [20].

On the other hand, voiding dysfunctions can be found in 5–20% of patient affected by endometriosis before any kind of pelvic surgery, and only a small number of these cases can be “anatomically” explained by localisation of endometriotic foci in the lower urinary tract [21, 22]. A few case series—where a urodynamic study was systematically performed before surgery for DIE—showed a functional bladder outflow obstruction in a significant number of patients [21, 23], suggesting that dysfunctional voiding is a common finding in the natural history of the disease [4]. Furthermore, a strong relationship between symptoms and localisation of endometriotic lesions has never been clearly established, on either the urological or gastrointestinal side, where symptoms frequently occur in women free of bowel nodules. In both cases, chronic inflammation, an aberrant immune response and hormonal changes, associated with genetic individual predisposition and environmental influences, may be determining factors in the pathogenesis of symptoms [24, 25].

After pelvic surgery, urinary symptoms are necessarily due to a combination of anatomical damage and pre-existing functional damage, which is part of the natural history of the disease.

Whilst the organic lesions of the hypogastric and pelvic plexus cannot be restored yet, it is conceivable that SNM therapy, which has been successfully used for both treatment of unsustained bladder contraction and dysfunctional voiding [26], may lead to an improvement of the functional damage.

Similarly, only a few gastrointestinal disorders can be explained by the presence of endometriotic rectal nodules, and cyclic inflammatory phenomena that lead to irritation of bowel mucosa have been proposed as a contributory cause of bowel dysfunctions, such as constipation, in patients suffering from endometriosis prior to surgery. Moreover, pre-existent gastrointestinal symptoms can be associated with de novo bowel disorders because of radical surgery and pelvic denervation [24]. Considering the good success of SNM on bowel dysfunctions, we cannot exclude a role of SNM in controlling gastrointestinal symptoms related to endometriosis.

A few case series about SNM in patients affected by endometriosis and treated with surgery are available.

Lavonius et al. described their experience with SNM on pain-related symptoms in four women previously treated with pelvic surgery for DIE. Three patients obtained a benefit from SNM, intended as a reduction of pain drugs assumption or a significant improvement in validated questionnaire score [27].

Nyangoh and colleagues retrospectively enrolled five patients with bladder acontractility and need for self-catheterisation due to pelvic surgery for DIE. After SNM and a 4-year follow-up, one patient stopped self-catheterisation because of a complete resumption of spontaneous micturition; a second patient reduced the daily catheters from 4 to 2, with post-voiding volume always < 100 ml [28].

In our series, we retrospectively analysed 13 patients referred to our centre in a 47-month time period, for persistent VS/Ds after surgery for DIE. All patients underwent a standardised SNM implant procedure, performed by a single surgical team. Nine patients obtained a clinical benefit on bladder sensibility and emptying, pain control or bowel management during the testing phase and underwent definitive implant.

All of our patients were treated with SNM after at least 2 years from the last pelvic surgery. According to different authors, spontaneous improvement of bladder function after pelvic surgery is possible up to 1–3 years after the surgery. Hence, it would appear logical to propose a SNM test for patients with VS/Ds lasting > 1 year [28]. Further studies need to be planned to understand if SNM can be useful to treat dysfunctional voiding in patients suffering from endometriosis in an earlier stage, when surgery is not even an option.

Considering the possible serious damage of pelvic surgery on neural pathways, it might be helpful to include neurophysiological studies as part of the diagnostic evaluation of patients before offering SNM as a potential therapeutic option. Furthermore, a recent study shows how a peripheral neuropathy is already present in > 30% of patients before surgery due to endometriosis inflammation and nerve involvement [23]. On the other hand, results regarding peripheral neuropathy should be interpreted with caution as electromyography explores innervation of pelvic-perineal muscles, which reflect lesions of the pudendal nerve and not of the autonomic nervous system. Moreover, PNTMLs do not reliably reflect the pudendal nerve functional status, since they measure the speed of nerve conduction, which involves the fastest nerve fibres, which are less susceptible to damage [29]. In our population, a bilateral pudendal neuropathy was present in two out of three patients that underwent PNTMLs. One patient did not respond to SNM. This finding could suggest an intact peripheral neural pathway might be needed to obtain a benefit from neuromodulation.

The small sample size, a lack of standardised diagnostic criteria and the retrospective nature of the study are limits that must be overcome to obtain strong data and predictive factors of SNM response in patients affected by endometriosis.

## Conclusions

VS/Ds and UDS findings after surgery for deep-infiltrating endometriosis should be explained by both anatomical damage of the inferior hypogastric/pelvic plexus and worsening of pre-existent functional damage which is part of the natural history of the disease.

On one hand, better topographic understanding of the autonomic pelvic network should help to prevent iatrogenic injury through the adoption of nerve-sparing surgical techniques to reduce postoperative autonomic dysfunction. On the other hand, the use of SNM to improve the voiding dysfunction and functional damage is certainly worth considering. Results on pelvic pain control and gastrointestinal disorders should not be underestimated. The results of our study are encouraging. Further studies with greater number of patients and standardised diagnostic evaluations are required to assess the efficacy of SNM for the control of VS/Ds after surgery for DIE.
